# Ameliorative effect of a standardized polyherbal combination in methotrexate-induced nephrotoxicity in the rat

**DOI:** 10.1080/13880209.2020.1717549

**Published:** 2020-02-21

**Authors:** Sanchit Sharma, Sanjula Baboota, Saima Amin, Showkat R. Mir

**Affiliations:** a Department of Pharmacognosy and Phytochemistry, School of Pharmaceutical Education and Research, Jamia Hamdard, New Delhi, India; b Department of Pharmaceutics, School of Pharmaceutical Education and Research, Jamia Hamdard, New Delhi, India

**Keywords:** Herbal combination, nephroprotection, xanthine oxidase, RSM, HPLC

## Abstract

**Context:**

Nephrotoxicity is a renal dysfunction that arises from direct exposure to environmental chemicals or as a side effect of therapeutic drugs. *Boerhaavia diffusa* Linn. (Nyctaginaceae), *Rheum emodi* Wall. Ex. Meissn. (Polygonaceae), *Nelumbo nucifera* Gaertn. (Nelumbonaceae) and *Crataeva nurvala* Buch-Ham. (Capparidaceae) are well-recognized medicinal plants of Indian traditional system of medicine used for kidney disorders.

**Objectives:**

The present investigation was undertaken to develop a chromatographically characterized polyherbal combination and to evaluate its nephroprotective activity.

**Materials and methods:**

Roots of *B. diffusa* and *R. emodi*, flowers of *N. nucifera* and stem bark of *C. nurvala* were extracted by decoction using 70% ethanol. Response surface methodology (RSM) was used for the optimization of extraction parameters. Polyherbal combinations with different doses (150–300 mg/kg) were tested against methotrexate-induced nephrotoxicity in Wistar rats.

**Results:**

The optimized extract contained 27% phenols and 15% flavonoids, which showed 75% 1, 1-diphenyl-2-picryl-hydrazyl (DPPH) scavenging potential. Based on the retention time of high-performance liquid chromatography (HPLC) analysis, 17 out of 122 constituents were found common in all extracts and combinations. Two combinations showed significantly higher (*p* ≤ 0.05) DPPH scavenging potential and xanthine oxidase inhibition. The half maximal inhibitory concentration (IC_50_) of the best combination for DPPH scavenging and xanthine oxidase inhibition were 80 and 74 µg/mL, respectively. Treatment of methotrexate-induced nephrotoxic rats with polyherbal combination significantly (*p* ≤ 0.05) improved the kidney function markers, oxidative stress markers and histological parameters.

**Discussion and conclusion:**

The developed combination was found to be effective in nephrotoxicity; it can be explored further for the management of drug-induced nephrotoxicity and other chronic kidney diseases.

## Introduction

The kidney carries out the essential excretory procedures in the human body, therefore becoming the primary target organ for different circulating toxins that can lead to nephrotoxicity. Nephrotoxicity is a renal dysfunction that arises from direct exposure to environmental chemicals or as a side effect of many drugs such as some nonsteroidal anti-inflammatory drugs, aminoglycoside antibiotics, anticancer drugs, etc. (Perazella 2018). Methotrexate, a folic acid antagonist, is widely used in the treatment of various malignancies and inflammatory diseases. However, nephrotoxicity is a substantial adverse effect of methotrexate therapy (Widemann 2006). The pathogenesis of methotrexate nephrotoxicity involves multiple pathways, including oxidative stress and inflammation (Devrim et al. 2005). Gentamycin, the commonly used broad-spectrum aminoglycoside antibiotic, causes nephrotoxicity in 30% of cases. Long-term exposure of gentamycin results in dysfunction of mitochondria and the formation of reactive oxygen species (ROS). Overproduction of ROS increases the oxidative burden, which causes inflammation and apoptosis in the proximal convoluted tubular kidney cells. Another primary reason for nephrotoxicity is the generation of free radicals in the kidney cortex that leads to dysfunction of renal proximal tubule cells. Clinically significant nephrotoxicity has been observed due to the administration of various other therapeutic drugs. Several agents have been used, with various degrees of success, to ameliorate or prevent methotrexate nephrotoxicity (Caglar et al. 2013).

Researchers have evaluated different approaches, including atrial natriuretic peptide, low dose dopamine, endothelin antagonists, loop diuretics, prostaglandin analogues, sodium bicarbonate and α-lipoic acid to manage kidney damage due to nephrotoxicity (Waikar and Bonventre 2009). However, the current treatment of kidney damage is still empirical. Therapeutic agents are used indiscriminately without considering the underlying aetiology of kidney damage. Though these approaches have shown favourable results in several experimental models, they have either failed to show consistent benefit or proved ineffective when used therapeutically. Due to the limitations of modern medicine, researchers are exploring medicinal plants that are used to treat patients having impaired renal functions.

*Boerhaavia diffusa* L. (Nyctaginaceae), known as punarnava in Sanskrit, is an herbaceous plant. Its name means that it ‘renews the body.’ As per the Ayurvedic literature, it is claimed to be rejuvenative for the urinary system (Rajpoot and Mishra 2011). In the Ayurvedic system, *B. diffusa* is used to improve the function of impaired kidneys. In oedematous conditions, it helps the healthy kidneys to expel the excess fluid out of the body very effectively (Sawardekar and Patel 2015). Various experimental studies have also illustrated its diuretic and possible nephroprotective effects against drug-induced renal damage (Pareta et al. 2011a). However, the exact mechanism of diuresis and nephroprotective potential has not been evaluated.

*Crataeva nurvala* Buch.-Ham (Capparidaceae) is a well-recognized medicinal plant of the Indian traditional system of medicine used for various therapeutic purposes since ancient times (Goyal et al. 2010). *C. nurvala* has been evaluated for the management of urolithiasis (Agarwal et al. 2010), obstructive uropathies, neurogenic bladder, chronic urinary infections (Cho et al. 2015) and kidney stone (Yadav et al. 2016). *In vivo* studies have proved that *C. nurvala* stem bark has a protective role against free radical-induced toxicity in experimental urolithiasis and hepatotoxicity (Sunitha et al. 2001).

*Rheum emodi* Wall. Ex. Meissn. (Polygonaceae) is extensively used in both Ayurvedic and Unani systems of medicine. It has been described as having diuretic, liver stimulant, purgative/cathartic and stomachic properties (Rehman et al. 2014). Its usefulness in kidney disorders has also been reported (Alam et al. 2005). Different extracts of *R. emodi* are said to possess significant nephroprotective effects (Takako et al. 1984).

*Nelumbo nucifera* Gaertn. (Nelumbonaceae) is a perennial and rhizomatous aquatic herb with slender, elongated, branched and creeping stem. This plant has been widely used as a component of traditional Chinese, Indian, Japanese, Thai and Korean medicines (Tungmunnithum et al. 2018). The whole plant is used as herbal medicine to cure diarrhoea, insomnia, fever, body heat imbalance and gastritis (Paudel and Panth 2015). In the traditional system of medicine, *N. nucifera* is widely used in the management of tissue inflammation. The flowers are valued as an antiemetic, poisoning antidote, diuretic and refrigerant. To the best of our knowledge, no work is reported related to renal protection by *N. nucifera*.

In the traditional Indian system of medicine, herbs and herbal combinations have been used for many years because of their historical heritage and mythological belief. As per traditional knowledge, any complex disease can be treated only by a multidrug approach, unlike the Western medicine, which generally involves single-drug therapy. A single herb contains multiple numbers of metabolites and the herbal combination is composed of various herbs. The above-selected plant materials have been used in kidney disorders. No single polyherbal combination has been developed or evaluated for preventive activity in nephrotoxicity. A review of the scientific literature showed the absence of any experimental data to justify the protective role of polyherbal combination in nephrotoxicity.

Taking leads from the available research, we aimed to develop polyherbal combinations and to evaluate their effect on methotrexate-induced nephrotoxicity in rats and to explore the possible mechanisms involved in reversing the renal damage.

## Materials and methods

### Plant materials and extract preparation

*B. diffusa* (roots), *R. emodi* (roots), *N. nucifera* (flowers) and *C. nurvala* (stem bark) were procured from M/s Universal Biotech, Delhi, India. Taxonomist Dr H. B. Singh, National Institute of Science Communication and Information Resources (NISCAIR), New Delhi, authenticated the collected plant materials. The voucher specimen of plant materials was deposited in the Department of Pharmacognosy, School of Pharmaceutical Education and Research, with voucher No. GM/SS/PP/FP-08 to 11. The plant samples were dried individually in a hot air oven at 50 °C for 72 h. The dried plant materials were coarsely powdered (passed through 20 mesh) and kept in air-tight containers until used. Individual plant materials were separately extracted by heating at 60 °C with ethanol-water (70:30 *v/v*) for 8 h. The hydroethanolic extract was concentrated under reduced pressure in a rotary evaporator (Buchi, Switzerland) for the complete removal of solvent. The residue was weighed for determination of extractive value and stored at 4 °C for further characterization.

### Determination of total phenolic and flavonoid content

Total phenolic and flavonoid content was determined for all the extracts. Folin-Ciocalteu and aluminium chloride (AlCl_3_) colorimetric methods were followed for the determination of total phenolic and flavonoid content, respectively (Khan et al. 2017). Standard gallic acid and rutin were used for the quantification of phenol and flavonoids, respectively. Different concentrations of standards ranging from 10–100 µg/mL were used for establishing the calibration curves and the obtained regression equations were used for the determination of total content. The amount of phenol and flavonoids in the extract were expressed as mg/g of gallic acid and rutin equivalent, respectively. Quantitative estimation was performed in triplicate (Khan et al. 2017).

### HPLC fingerprinting of hydroalcoholic extract

Each dried extract (100 mg) was dissolved separately in 20 mL of high-performance liquid chromatography (HPLC) grade methanol (5 mg/mL). Prepared solutions were vortexed, sonicated, filtered and used for analysis. The chromatographic profiling was carried out with the use of HPLC system (Shimadzu, JAPAN) equipped with a ultraviolet (UV) detector, a gradient pump and the CLASS-VP software for data acquisition and processing. Filtered extract (10 µL) solution was injected and allowed to separate in the gradient elution mode using a Hypersil C18 (5 µ particle size) column (150 × 4.6 mm; Phenomenex, Torrance, CA, USA). Water with 0.5% (*v/v*) formic acid (A) and acetonitrile (B) were used as a mobile phase in a gradient elution programme: from 0 to 5 min, 80% A; from 5 to 12 min, 60% A; from 12 to 20 min, 40% A; from 20 to 30 min, 10% A and further it was returned to the initial composition of phase. The flow rate of mobile phase was set to 1 mL/min and a total run time of 30 min. The UV-Visible detector was set at 254 nm to record the chromatograms. For the determination of the quality of the extract, the total number of peaks were identified.

### Determination of antioxidant activity

The antioxidant potential of each extract was determined by the 1, 1-diphenyl-2-picryl-hydrazyl (DPPH) assay. The potential of extract to scavenge the free radicals were expressed as equivalent to antioxidant potential. Accurately weighed 10mg of each prepared extract was suspended separately in 70% methanol and vortexed for 2 min, followed by sonication for 25 min. Further, it was centrifuged at 1000 rpm for 10 min and the supernatant was used for analysis. The prepared extract stock solution was diluted with 70% methanol. Different concentrations of extract solution (10, 20 and 40 µg/mL) were mixed with 1 mL of a methanolic solution of DPPH (0.5 mM) and incubated for 30 min in the dark at room temperature. After complete incubation, absorbance was recorded at 517 nm using a UV-visible spectrophotometer (Dudonné et al. 2009). For the comparison of the antioxidant potential of extract, ascorbic was used as a standard.

### Statistical optimization of extraction parameters

For the optimization of extraction parameters, response surface methodology (RSM) was deployed. It is a collection of statistical techniques for designing experiments, building models, evaluating the effects of factors and searching for the optimum conditions. The RSM method determines the Taylor expansion curve surface that defines the response in terms of yield and antioxidant potential of extract (Alam et al. 2015). Extraction time (h), drug solvent ratio (*v/v*) and the alcohol-water ratio (*v/v*) were selected as parameters for optimization experiments. The effect of each parameter was evaluated initially using one factorial design. The selected levels for extraction time were 5 (−1), 6 (0) and 7 h (+1), for drug-solvent ratio (% *v/v*) were 5 (−1), 8.75 (0) and 12.5% (+1) and for alcohol-water ratio (% *v/v*) were 55 (−1), 62.5 (0) and 70% (+1), respectively. Different drug solvent ratios and alcohol-water ratios were screened for their maximum yield by keeping extraction time at a constant level 8 h. A 17 run experimental design having three central points were made according to Box-Behnken’s response surface design with above-selected parameters using Design Expert 8.0.1.7 software (Statease Inc., Minneapolis, MN, USA). Responses were measured in terms of antioxidant activity and extractive value (% yield). The point prediction tool of the software determined an optimum value of the factors for the production of maximum extractive value having high antioxidant activity (Alam et al. 2015).

### Determination of xanthine oxidase (XO) inhibition

XO inhibitory activity was measured as described in the manufacturer’s protocol of EnzyChrom^TM^ Xanthine Oxidase Assay Kit (BioAssay Systems, Hayward, CA, USA) as per a previously developed method (Bustanji et al. 2011). The substrate and the enzyme solutions were prepared immediately before use. The absorption of the reaction mixture was recorded at 570 nm immediately and after 20 min incubation at room temperature and the initial rate was calculated. The extract, dissolved in 70% methanol and diluted with sodium phosphate buffer, was incorporated in the enzyme assay to assess its inhibitory activity at different concentrations ranging from 50–300 μg/mL. All tests were run in triplicate. Inhibition percentages are the mean of three observations. A negative control (blank; 0% XO inhibition activity) was carried out following the same procedure without the extract. Allopurinol was used as a positive control in the assay mixture. The XO inhibitory activity was expressed as the percentage inhibition and was calculated as follows:

$$\mathrm{Percentage}\;of\;\mathrm{xanthine}\;\mathrm{oxidase}\;\mathrm{inhibition}=\left(Abs_{\mathrm{control}}-\;Abs_{\mathrm{sample}}\right)\times 100/Abs_{\mathrm{control}}$$

### Development of polyherbal combination

The extracts obtained by using optimized extraction parameters were used for preparing polyherbal combinations. A randomized block design was used to develop the polyherbal combination. The experiment used a randomized block design with four plant extracts at a fixed concentration and three replications to test the XO inhibition potential and DPPH scavenging activity of the polyherbal combination. A combination of these extracts were dissolved in a solvent. The concentration used for these combinations were 4, 3 and 2 mg/mL, respectively. This concentration was mixed separately with the same volume of solvent to achieve a final concentration of 1 mg/mL for each extract.

Further, the stock solution was diluted and used for testing purposes. A total of 10 combinations was subjected to evaluation. Polyherbal combination from randomized block design was ABCD, ABC, BCD, ABD, ACD, AB, AC, AD, BD and CD where A, B, C and D denote *B. diffusa*, *C. nurvala*, *N. nucifera* and *R. emodi*, respectively. The combinations were screened for XO inhibition potential and DPPH scavenging activity. For validation of the statistical model, different concentrations of one combination were tested for the selection of the best combination. The best combinations of the extract, which showed maximum XO inhibition and DPPH scavenging potential were selected for further *in vivo* evaluation.

The developed polyherbal combinations were characterized through HPLC fingerprinting. The established chromatographic conditions used for chromatographic profiling of extract were also used for the analysis of polyherbal combinations. The detection of ingredients of combinations was done at 254 nm. A total of 106 metabolites were detected.

### *In vivo* renal protection assay

#### Animals and treatment schedule

Ten-week-old male Wistar rats weighing 150–200 g were used after one week of proper acclimatization to the animal house environmental conditions (12 h light/dark cycle and 25 ± 2 °C temperature) with free access to standard rodent chow and water. All experimental procedures were carried by following the stringent guidelines of the Committee for the Purpose of Control and Supervision of Experiments on Animals, New Delhi, India (Registration No.: 173/GO/RE/S/2000/CPCSEA). The experimental protocol was approved by the Institutional Animal Ethical Committee of Jamia Hamdard, New Delhi, India (Approval No. 1206). From randomized block design, two best combinations (Combination ABCD: Combination of four extracts and Combination ABD: Combination of three extracts) were selected for *in vivo* evaluation.

Animals were randomly divided into nine groups (six animals each). The study was carried out for eight days. Group, I served as the control group that received a normal diet. Group II–VI were administered methotrexate 7 mg/kg/day (intraperitoneally) for the last three days of the study period to induce nephrotoxicity (Morsy et al. 2013). Group II animals received only a normal diet for the whole study period along with methotrexate treatment for the last three days to serve as toxic control. Group III and IV received combination ABCD with a dose of 200 and 300 mg/kg, respectively, for eight days. Group V and VI received combination ABD with a dose of 150 and 250 mg/kg, respectively, for eight days. Group VII received Ayurvedic marketed combination (Neeri KFT, Aimil Pharmaceutical Ltd, India) with a dose of 100 mg/kg. Group VIII and IX received combination ABCD and ABD with a dose of 200 and 150 mg/kg, respectively, for eight days, but no methotrexate treatment served as sham control of respective combinations. Animals of all groups received equivalent volumes of the vehicles. Rats were sacrificed 24 h after the last methotrexate injection, and blood samples were collected and centrifuged at 3000 *g* for 10 min to obtain clear serum.

### Evaluation of biochemical parameters

Serum creatinine, urea, uric acid, protein, albumin, globulin levels in serum were quantified by using commercially available kits according to their manufacturers’ guidelines (Span Diagnostics Ltd, Surat, India). Oxidative markers such as SGOT and SGPT were analyzed using a fully automated clinical chemistry analyzer (Chem 5X, Erbachem, Mannheim, Germany). Catalase (CAT) and malondialdehyde (MDA) were measured spectrophotometrically. As quantitative estimation of MDA depends on the formation of MDA as an end product of lipid peroxidation, which reacts with thiobarbituric acid, which produces a pink chromogen thiobarbituric acid reactive substance (TBARS), TBARS was measured spectrophotometrically at 532 nm (Ohkawa et al. 1979). Catalase estimation is based on the production of chromic acetate from dichromate and glacial acetic acid in the presence of hydrogen peroxide. Colorimetrically, chromic acetate was measured at 570 nm. Under assay condition, the amount of enzyme was expressed as equivalent to 1 μmol H_2_O_2_ per min (Sinha 1972). Renal tumor necrosis factor alpha (TNF-α) assay was performed with rat TNF-α ELISA kit (RayBiotech, Inc., Peachtree Corners, GA, USA) according to supplier’s instructions. For determination of oxidative stress markers in the kidney, excised kidneys were immediately frozen in liquid nitrogen and stored at -80 °C. Before analysis, frozen kidneys were homogenized in cold 50 mM phosphate buffer (pH 7.4).

Oxidative stress was directly measured by determining ROS in kidney homogenate by using florescent dye, namely 2, 7-dichlorofluorescein diacetate (H2-DCFH-DA). The stored kidney was thawed at room temperature and then washed with phosphate buffer saline. Further, it was homogenized and the cell suspension was prepared in Hanks' balanced salt solution. After that, 10 µM of DCFH-DA was added in cell suspension and incubated for 30 min at 37 °C. Reading was measured at Ex/Em = 485/530 nm in endpoint mode (Cary Eclipse, Varian, Agilent Technologies, Palo Alto, CA, USA; Chester et al. 2017). The fluorescence intensity was recorded and compared with different samples against normal control.

### Evaluation of histological parameters

For histological examination, the longitudinal part of the left kidney was excised from each animal. Renal tissue specimens were set for seven days in 10% neutral formalin buffered. Fixed tissue samples were embedded in paraffin, sectioned and stained with haematoxylin and eosin for histological examination. Using light microscopy, three sections of each group of animals were subjected to microscopic study. Changes in renal structure have been classified as mild, moderate, or severe. Scores +, ++ and +++ were assigned for mild, moderate and severe levels, revealing less than 25, 50 and 75% histopathological changes of total examined fields, respectively.

### *In silico* screening for XO inhibition activity

To predict the binding affinity of plant constituents, molecular modelling studies with bioactive constituents were accomplished. Boeravinone of *B. diffusa*, emodin of *R. emodi*, lupeol of *C. nurvala,* and armepavine of *N. nucifera* were selected for docking studies with XO proteins by the MOPAC6 software package (Stewart Computational Chemistry, Colorado Springs, CO, USA). XO enzyme with 1333 AA protein (Accession: NP_000370.2 GI: 91823271) was downloaded from protein data bank and processed with MOPAC2009 software package. All the docking calculations were achieved on different protein models. In AutoDock tools, solvation parameters, essential hydrogen atoms and Kollman united atom type charges were added. The auto grid programme was employed for the generation of affinity (grid) maps of × Å grid points and 0.375 Å spacing. Vander Waals and electrostatic terms were generated by AutoDock parameter set and distance-dependent dielectric functions, respectively. Simulations of docking were executed using Lamarckian genetic algorithm and Solis and Wets local search method (Solis and Wets 1981). Orientation, initial position and torsions of the ligand molecules have been randomly selected. During docking, all rotatable torsions were released. A translational step of 0.2 Å was used, whereas five quaternion and torsion steps were utilized in each search. In each docking experiment, two different runs were set, and it was terminated after the assessment of a maximum 2,50,000 Kcal energy was reached. The structure of molecules in mol format was generated in the CDX format using the tool ChemDraw Ultra 7.0.1 (CambridgeSoft Corporation, PerkinElmer, Waltham, MA, USA) and transformed to input ligand format (pdb) for docking by OpenBabel version 2.3.2 (O'Boyle et al. 2011).

### Statistical analysis

Data were expressed as mean ± standard error mean (SEM). Two-way analysis of variance (ANOVA) followed by ‘Bonferroni post-tests’ (Graph Pad, San Diego, CA, USA) was used for statistical analysis for the determination of significant differences. Normal groups were compared against toxic control, while the other treatment groups were compared against toxic control. *p* Values <0.05 were considered as statistically significant.

## Results

### Preparation of extracts and their characterization

**Figure 1.**
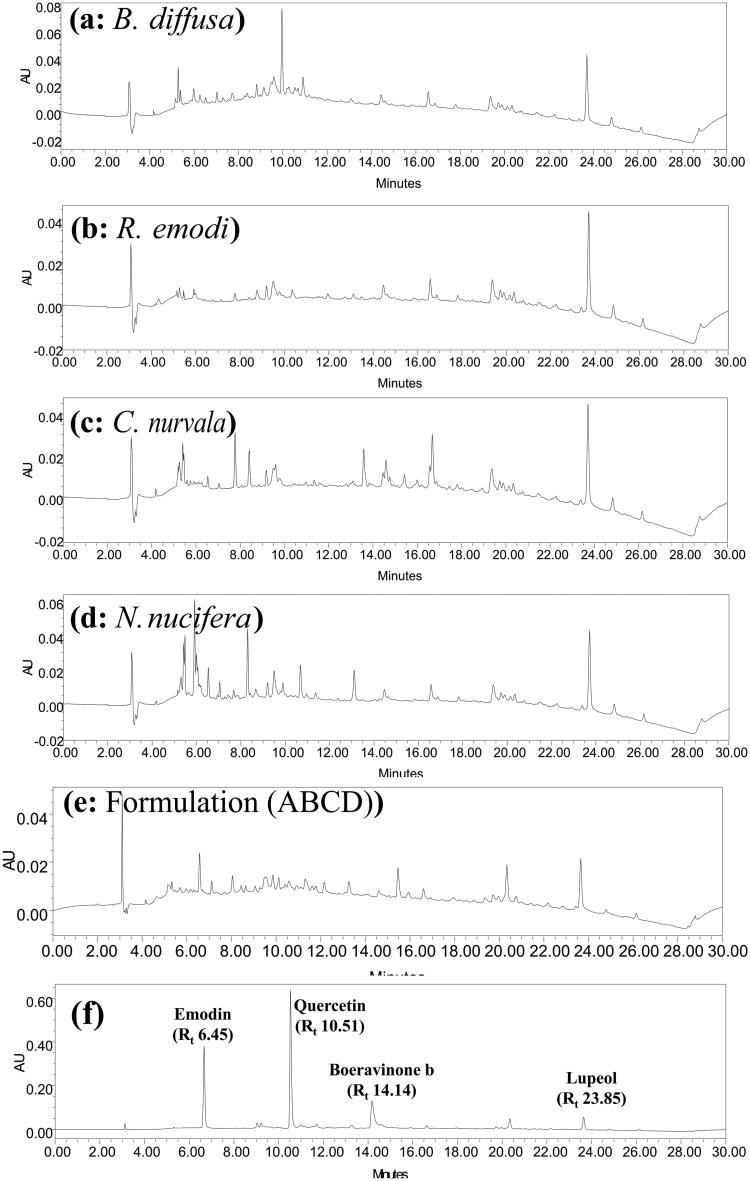
HPLC chromatogram of hydroalcoholic extract of (a) *B. diffusa*, (b) *R. emodi*, (c) *C. nurvala*, (d) *N. nucifera*, (e) optimized combination (ABCD) and (f) standards recorded at 254 nm.

The dried plant materials viz; *B. diffusa* (roots), *R. emodi* (roots), *N. nucifera* (flowers) and *C. nurvala* (stem bark), were extracted separately with 70% ethanol at 60 °C for 8 h by reflux. It was followed by evaporation under reduced pressure to yield residues with 15.25, 24.64, 12.61 and 14.35% *w/w* extractive value, respectively. Figure 1 shows the HPLC chromatogram of hydroalcoholic extract recorded at 254 nm. HPLC analysis resulted in the detection of 47, 53, 52 and 104 metabolites in *B. diffusa*, *R. emodi*, *N. nucifera* and *C. nurvala*, respectively. Chromatograms indicated the presence of a total of 122 metabolites in four plant extracts out of which 17 metabolites were found in common. Major abundant metabolites recorded in *B. diffusa* were eluted at R_t_ 5.153 (6.5%), R_t_ 3.428 (5.56%), R_t_ 9.94 (4.14%) and R_t_ 9.543 (3.96%). Metabolites eluted at R_t_ 5.153 (8.37%), R_t_ 3.428 (7.3%), R_t_ 1.942 (5.75%), R_t_ 2.769 (4.98%) and R_t_ 3.07 (4.91%) were the major metabolites found in *R. emodi*. Major abundant metabolites recorded in *N. nucifera* were eluted at R_t_ 5.192 (8.22%), R_t_ 3.428 (6.47%), R_t_ 1.942 (5.21%), R_t_ 2.746 (4.26%), R_t_ 3.075 (4.24%) and R_t_ 5.869 (3.79%). Similarly, metabolites eluted at R_t_ 23.707 (3.24%), R_t_ 5.192 (2.94%), R_t_ 19.362 (2.31%), R_t_ 15.42 (2.13%) and R_t_ 15.993 (1.83%) were the major metabolites found in *C. nurvala*. All the metabolites of four plant extracts with their retention time is shown in Table 1. Four markers were identified in plant extracts by matching the chromatogram of standard compounds under the same chromatographic conditions. Metabolite eluting at R_t_ 10.51 min was found in all extracts and identified as quercetin. Boeravinone B, a specific marker was eluted at R_t_ 14.14 min and detected in the hydroalcoholic extract of *B. diffusa*. Similarly, emodin, an anthraquinone glycoside present in *R. emodi,* was identified by matching peak eluting at R_t_ 6.451 min. A pentacyclic triterpenoid, lupeol found to be eluting at R_t_ 23.85 min, was present only in the hydroalcoholic extract of *C. nurvala*.

**Table 1.** HPLC fingerprinting of hydroalcoholic extract and polyherbal combination.

R_t_ (min)	*B. diffusa*	*R. emodi*	*N. nucifera*	*C. nurvala*	Combination (ABCD)
1.942	+	+	+	+	+
2.746	−	−	+	+	+
2.769	+	+	−	−	+
3.075	+	+	+	+	+
3.428	+	+	+	+	+
4.184	+	+	+	+	+
4.34	−	+	−	−	+
5.153	+	+	−	−	+
5.192	−	−	+	+	+
5.303	+	+	+	+	+
5.39	+	−	−	+	+
5.421	−	−	+	−	+
5.451	−	+	+	+	+
5.601	+	−	+	+	+
5.618	−	+	+	−	+
5.676	+	+	−	+	+
5.869	+	+	+	+	+
5.986	+	+	+	+	+
6.06	−	−	+	−	+
6.12	−	−	+	+	+
6.212	+	+	+	+	+
6.271	−	−	+	+	+
Emodin (R_t_ 6.415)	−	+	−	−	+
6.523	+	+	+	+	+
6.684	−	−	+	−	−
6.755	+	+	+	−	+
6.874	−	+	+	+	+
7.026	+	+	+	+	+
7.14	−	+	+	−	+
7.254	−	−	+	+	+
7.316	+	+	−	+	+
7.427	−	−	+	−	+
7.498	+	+	+	−	+
7.642	−	−	+	+	+
7.726	+	+	+	+	+
7.869	−	−	+	−	−
7.985	+	−	−	−	+
8.073	+	+	+	+	+
8.214	+	+	−	+	+
8.305	+	+	+	−	+
8.407	+	−	−	+	+
8.639	−	+	+	+	+
8.822	+	+	−	+	+
8.936	+	−	+	−	+
9.062	−	−	+	+	+
9.15	+	+	+	+	+
9.484	+	+	+	+	+
9.593	+	−	−	+	+
9.776	−	+	+	+	+
9.896	−	+	+	−	+
9.94	+	+	+	+	+
10.196	+	+	+	−	+
10.262	+	−	−	−	+
10.354	+	+	+	+	+
Quercetin (R_t_ 10.51)	+	+	+	+	+
10.674	+	−	+	+	+
10.809	−	+	−	+	+
10.909	+	+	+	+	+
11.182	+	+	−	+	+
11.342	−	−	+	+	+
11.591	+	−	−	+	+
11.695	+	+	−	+	+
11.95	−	+	−	+	+
12.061	+	−	−	+	+
12.242	−	−	−	+	−
12.499	−	−	−	+	−
12.702	+	0.9	−	+	+
12.801	−	−	−	+	−
12.988	−	−	−	+	−
13.079	+	+	+	+	+
13.22	−	−	−	+	−
13.386	−	+	−	+	−
13.579	−	−	−	+	+
13.835	−	−	−	+	−
13.937	−	−	−	+	+
Boeravinone (R_t_ 14.14)	+	−	−	−	+
14.452	−	+	+	+	+
14.585	−	+	−	+	+
14.751	−	−	−	+	+
14.932	−	−	−	+	+
15.095	−	−	−	+	−
15.422	−	−	−	+	+
15.696	−	−	−	+	−
15.993	−	−	−	+	+
16.175	−	−	−	+	−
16.558	+	+	+	+	+
16.678	−	−	−	+	+
16.856	−	−	−	+	+
17.01	−	−	−	+	−
17.229	−	−	−	+	−
17.454	−	−	−	+	+
17.803	−	−	−	+	+
18.004	−	−	−	+	+
18.411	−	−	−	+	+
18.49	−	−	−	+	+
18.939	−	−	−	+	+
19.362	+	+	+	+	+
19.724	−	+	+	+	+
19.881	−	+	−	+	+
20.138	−	−	−	+	+
20.335	−	+	−	+	+
20.629	−	−	−	+	−
20.758	−	−	−	+	+
20.996	−	−	−	+	+
21.302	−	−	−	+	−
21.458	−	−	−	+	+
21.703	−	−	−	+	+
22.107	−	−	−	+	+
22.253	−	−	−	+	−
22.899	−	−	−	+	−
23.373	−	−	−	+	+
23.707	+	+	+	+	+
Lupeol (R_t_ 23.85)	−	−	−	+	+
24.376	−	−	−	+	+
24.823	−	+	+	+	+
25.034	−	−	−	+	−
25.373	−	−	−	+	−
25.611	−	−	−	+	+
26.159	−	−	−	+	+
28.751	+	+	+	+	−
29.195	+	−	−	−	+
Total (122)	47	53	52	104	105

### Total phenolic and flavonoid content of the prepared extracts

**Figure 2.**
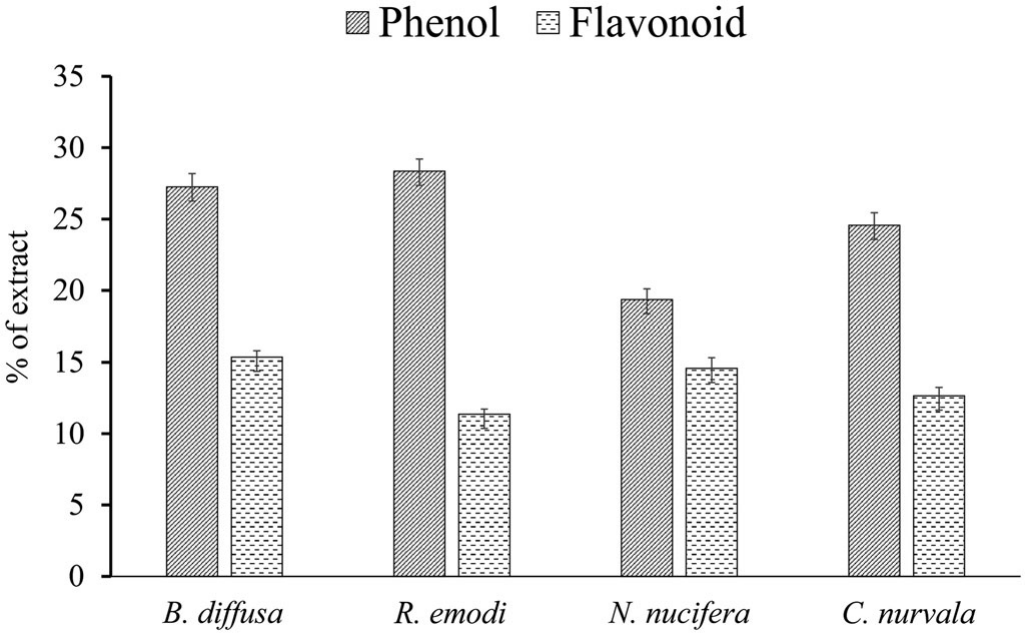
Phenolic and flavonoid content hydroalcoholic extract of different plants.

The phenolic contents of the extracts was calculated from the calibration curve (coefficient of correlation (*R*^2^) = 0.998), and it was expressed as gallic acid equivalents per 100 g of the crude extract. In the same way, total flavonoid content was measured from the calibration curve of rutin (*R*^2^ = 0.999) and it was expressed as rutin equivalents per 100 g of extract. Phenol and flavonoid contents of the extract are shown in Figure 2 as a percentage of the total extract. The hydroxyl group present in phenolic compounds and flavonoids is responsible for the antioxidant activity through redox reaction (Soobrattee et al. 2005). Hydroxyl groups facilitate scavenging of free radicals and thus total phenolic content is used as a basis for rapid screening of antioxidant activity of plant extract. The antioxidant activity of flavonoids, including flavones, flavonols and condensed tannins, is attributed to the existence of free OH groups, particularly 3-OH groups. Plant flavonoids and phenolics have been reported to be responsible for both *in vitro* and *in vivo* antioxidant activity (Shimoi et al. 1996; Geetha et al. 2003).

### Antioxidant potential of extracts

The hydroalcoholic extract of selected plants were found to be rich in secondary metabolites with antioxidant activity owing to the presence of phenolics and flavonoids. Because of the ease of response, radical DPPH is frequently used to determine any plant extract’s free radical scavenging activity. The maximum DPPH scavenging activity was recorded for *R. emodi* (78.6%), followed by *B. diffusa* (74.25%), *C. nurvala* (68.56%) and *N. nucifera* (64.36%), while that of the control, ascorbic acid, was 83.42%. The DPPH scavenging potential of each extract was increased upon concentration increment. Flavonoids are extremely potent scavengers of most oxidizing molecules, including singlet oxygen and other free radicals. Flavonoids suppress reactive oxygen formation, scavenge reactive species, up-regulate antioxidant defences and chelate free-radical in trace elements (Agati et al. 2012). Similarly, phenolic metabolites confer oxidative stress tolerance in plants (Shukla et al. 2009).

### Statistical optimization for preparation of the best extract

#### Analysis of variance and validation of the model

Three significant parameters that affect the extraction efficiency and antioxidant potential, namely extraction time, drug-solvent ratio and alcohol-water ratio, were optimized using Box-Behnken’s Design. Four selected plant materials were extracted under specified conditions separately. The yield of extract and its antioxidant activity were the two responses considered for optimization. The experimental and the predicted yield values of each plant extract are presented in Table 2. Table 3 shows the experimental and predicted antioxidant activities of each extract prepared. Results were the average of three independent assays.

**Table 2.** The predicted and experimental value for % yield of extraction trials carried out by Box-Behnken’s response surface design for selected plant materials.^a^

Run	A	B	C	*N. nucifera*		*B. diffusa*		*C. nervala*		*R. emodi*	
				Actual	Predicted	Actual	Predicted	Actual	Predicted	Actual	Predicted
1	6.00	12.50	70.00	19.8	18.34	19.8	18.34	13.64	13.99	27.01	25.69
2	7.00	12.50	62.50	15.24	15.50	15.24	15.50	14.00	14.08	30.25	30.02
3	6.00	5.00	70.00	11.66	11.73	11.66	11.73	15.20	15.12	21.50	23.51
4	7.00	8.75	70.00	11.84	13.03	11.84	13.03	10.76	10.41	16.80	19.90
5	5.00	5.00	62.50	8.54	8.28	8.54	8.28	15.12	15.34	15.60	18.58
6	6.00	8.75	62.50	10.65	10.69	10.65	10.69	13.89	14.38	18.90	20.79
7	7.00	5.00	62.50	11.56	10.29	11.56	10.29	18.80	18.31	31.10	30.62
8	6.00	8.75	62.50	10.7	10.69	10.7	10.69	14.88	14.66	30.70	29.13
9	5.00	8.75	70.00	8.98	9.17	8.98	9.17	14.80	14.23	24.60	23.95
10	6.00	8.75	62.50	10.68	10.69	10.68	10.69	14.39	14.25	19.40	15.43
11	6.00	5.00	55.00	8.95	10.41	8.95	10.41	17.01	17.15	28.95	31.77
12	6.00	8.75	62.50	10.7	10.69	10.7	10.69	14.00	14.57	28.50	27.99
13	6.00	12.50	55.00	12.6	12.53	12.6	12.53	14.40	14.42	25.60	24.78
14	5.00	8.75	55.00	7.8	6.61	7.8	6.61	14.40	14.42	25.60	24.78
15	6.00	8.75	62.50	10.7	10.69	10.7	10.69	14.40	14.42	25.60	24.78
16	5.00	12.50	62.50	10.54	11.81	10.54	11.81	14.40	14.42	25.60	24.78
17	7.00	8.75	55.00	8.65	8.46	8.65	8.46	14.50	14.42	25.60	24.78

A: extraction time (hour); B: drug solvent ratio (*v/v*); C: alcohol water ratio (*v/v*).

^a^Levels for extraction time are 5 h (−1), 6 h (0) and 7 h (+1); for drug solvent ratio (%*v/v*) are 5 (%*v/v*) (−1), 8.75 (%*v/v*) (0) and 12.5 (%*v/v*) (+1); for alcohol water ratio (%*v/v*) are 55 (%*v/v*) (−1), 62.5 (%*v/v*) (0) and 70 (%*v/v*) (+1).

**Table 3.** The predicted and experimental value for antioxidant potential (% of DPPH free radical scavenged) of extraction trials carried out by Box-Behnken’s response surface design for selected plant materials.^a^

Run	A	B	C	*N. nucifera*		*B. diffusa*		*C. nervala*		*R. emodi*	
				Actual	Predicted	Actual	Predicted	Actual	Predicted	Actual	Predicted
1	6.00	12.50	70.00	80.65	76.50	80.65	76.50	68.67	71.48	84.99	84.63
2	7.00	12.50	62.50	48.65	50.97	48.65	50.97	77.98	78.57	81.58	81.14
3	6.00	5.00	70.00	70.65	69.48	70.65	69.48	74.89	73.30	82.58	83.02
4	7.00	8.75	70.00	68.98	70.81	68.98	70.81	76.58	72.77	82.69	83.05
5	5.00	5.00	62.50	47.56	45.24	47.56	45.24	66.02	63.66	77.56	77.30
6	6.00	8.75	62.50	65.46	65.50	65.46	65.50	77.89	77.75	68.90	68.72
7	7.00	5.00	62.50	51.26	50.60	51.26	50.60	81.65	81.12	78.89	79.07
8	6.00	8.75	62.50	65.39	65.50	65.39	65.50	71.89	73.58	83.92	84.19
9	5.00	8.75	70.00	71.65	75.14	71.65	75.14	78.56	77.96	75.58	76.20
10	6.00	8.75	62.50	65.45	65.50	65.45	65.50	59.65	63.45	65.99	65.81
11	6.00	5.00	55.00	51.26	55.41	51.26	55.41	74.52	72.09	74.11	74.29
12	6.00	8.75	62.50	65.65	65.50	65.65	65.50	80.65	82.62	85.60	84.98
13	6.00	12.50	55.00	55.65	56.82	55.65	56.82	75.90	74.03	79.80	79.64
14	5.00	8.75	55.00	54.26	52.43	54.26	52.43	75.90	74.03	78.90	79.64
15	6.00	8.75	62.50	65.56	65.50	65.56	65.50	72.50	74.03	79.89	79.64
16	5.00	12.50	62.50	52.65	53.31	52.65	53.31	72.60	74.03	79.80	79.64
17	7.00	8.75	55.00	63.25	59.76	63.25	59.76	72.60	74.03	79.80	79.64

A: extraction time (h); B: drug solvent ratio (*v/v*); C: alcohol water ratio (*v/v*).

^a^Levels for extraction time are 5 h (−1), 6 h (0) and 7 h (+1); for drug solvent ratio (%*v/v*) are 5 (%*v/v*) (−1), 8.75 (%*v/v*) (0) and 12.5 (%*v/v*) (+1); for alcohol water ratio (%*v/v*) are 55 (%*v/v*) (−1), 62.5 (%*v/v*) (0) and 70 (%*v/v*) (+1).

The experimental results were modelled with second order polynomial equation with quadratic model to explain the dependence of *B. diffusa* extraction for both yield value and antioxidant activities on the different factors:

*B. diffusa* extract as yield: 12.52 – 1.16A – 0.65B – 0.63C   – 4.7AB + 2.2AC + 1.20BC + 2.92A^2^ – 0.74B^2^ – 2.38C^2^

*B. diffusa* extract as antioxidant activity: 74.98 – 4.78A   – 0.66B – 3.14C – 5.27AB + 3.43AC + 1.41BC   + 1.79A^2^ + 0.27B^2^ – 5.63C^2^

The experimental results were modelled with two factor interaction (2FI) and quadratic polynomial equation model to explain the dependence of *R. emodi* extraction for yield value and antioxidant activities, respectively, on the different factors:

*R. emodi* extract as yield: 24.78 + 0.18A – 3.08B + 5.09C   – 1.98AB – 0.93AC + 1.19BC

*R. emodi* extract as antioxidant activity: 74.94 – 0.87A   + 0.075B + 4.31C + 0.88AB + 3.42AC + 5.27BC   + 2.66A^2^ + 0.66B^2^ – 4.98C^2^

For the statistical experimental results of *N. nucifera* were modelled with second order polynomial equation with quadratic model to explain the dependence of its extraction for both yield value and antioxidant activities on the different factors:

*N. nucifera* extract as yield: 10.69 + 1.43A + 2.18B + 1.79C   + 0.42AB + 0.50AC + 1.12BC – 1.58A^2^ + 2.36B^2^   + 0.21C^2^

*N. nucifera* extract as antioxidant activity: 65.50 + 0.75A   + 2.11B + 8.44C – 1.92AB – 2.92AC + 1.40BC   – 7.74A^2^ – 7.73B^2^ + 6.78C^2^

The experimental results were modelled with 2FI and quadratic polynomial equation model to explain the dependence of *C. nurvala* extraction for antioxidant activity and yield, respectively, on the different factors:

*C. nurvala* extract as antioxidant activity: 74.03 + 1.64A   – 1.00B + 3.32C – 1.90AB – 5.41AC + 6.26BC

*C. nurvala* extract as yield: 14.42 – 1.15A – 0.64B + 0.81C   – 1.20AB – 0.67AC – 0.65BC – 0.20A^2^   – 0.82B^2^ + 1.45C^2^

**Figure 3.**
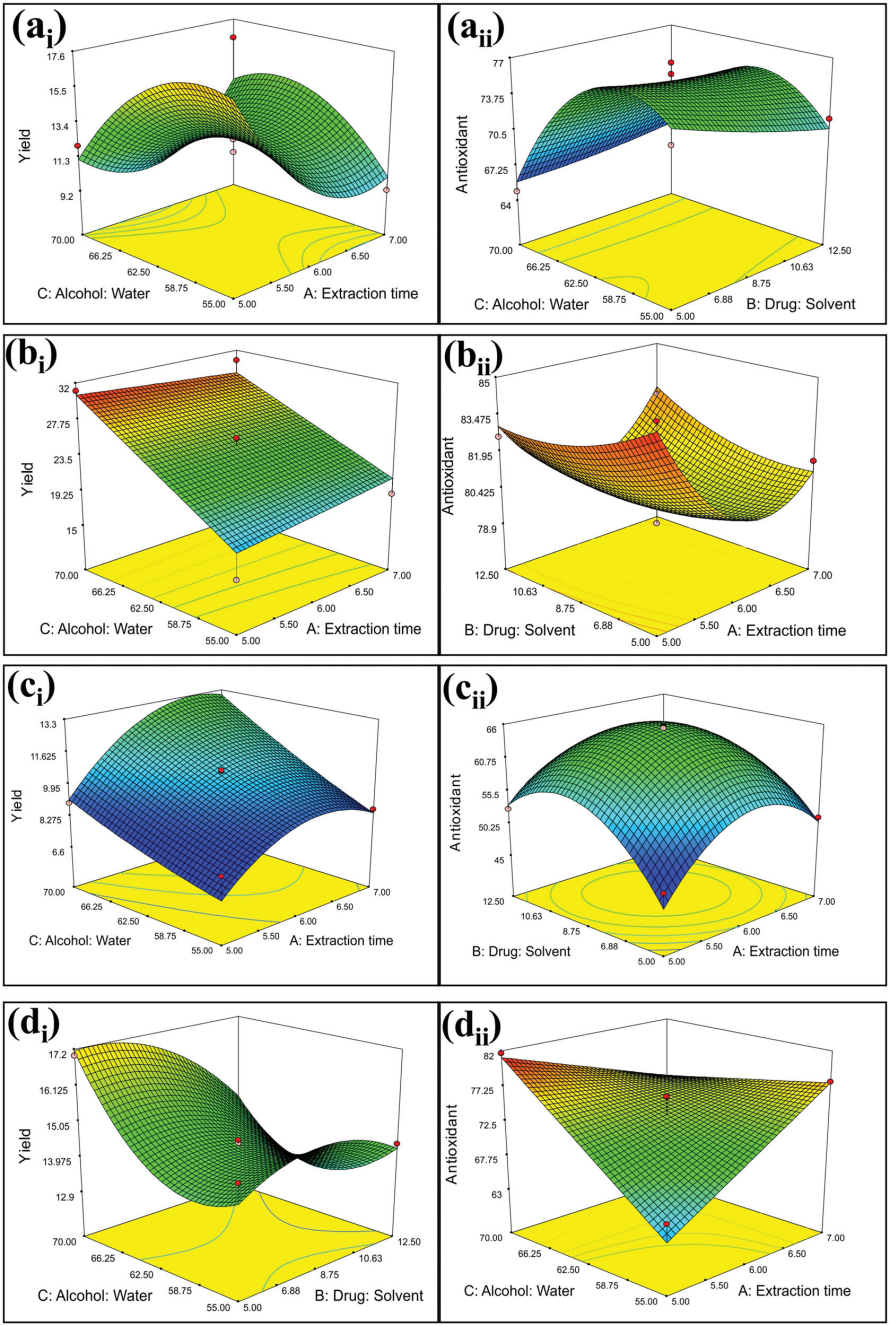
Response surface plots showing relative effect of two extraction parameters for (a) *B. diffusa* (*a*_i_: yield and *a*_ii_: antioxidant activity); (b) *R. emodi* (*b*_i_: yield and *b*_ii_: antioxidant activity); (c) *N. nucifera* (*c*_i_: yield and *c*_ii_: antioxidant activity) and (d) *C. nurvala* (*d*_i_: yield and *d*_ii_: antioxidant activity); but other parameters were kept at constant levels.

Where A, B and C were the coded values for extraction time (*h*), drug solvent ratio (% *w/v*), and alcohol water ratio (% *v/v*), respectively. Statistical analysis of results was performed to determine the significant differences. The 3 D response surface plots of extraction of selected plant materials based on their yield value and antioxidant activity as response are represented in Figure 3.

ANOVA analysis for yield value and antioxidant activities showed that the regression model was significant, and the lack of fit was insignificant for each plant (Table 4). The fit of the models evaluated through an *R*^2^. The regression equations obtained indicated an adequate adjustment of the quadratic model to the experimental data. The closer the values of *R*^2^ to 1, the better the model that explains the variability between the experimental and the model predicted values.

**Table 4.** Statistical analysis of the models for antioxidant potential obtained from response surface methodology for extraction of selected plant.

Parameters	*N. nucifera*		*B. diffusa*		*C. nervala*		*R. emodi*	
	Yield	Antioxidant	Yield	Antioxidant	Yield	Antioxidant	Yield	Antioxidant
Regression								
Sum of square	119.33	1346.7	119.33	1346.7	39.75	406.01	308.36	445.7
Df	9	9	9	9	9	9	6	9
Mean squares	13.26	149.64	13.26	149.64	4.42	67.67	51.39	49.52
F value	8.83	13.13	8.83	13.13	20.01	9.39	8.8	145.59
*p* Value	0.0045	0.0013	0.0045	0.0013	0.0003	0.0012	0.0016	0.0001
Residual								
Sum of square	10.51	79.8	10.51	79.8	1.54	72.06	58.37	2.38
Df	3	7	3	7	7	10	10	7
Mean square	3.50	11.40	3.50	11.40	0.22	7.21	5.84	0.34
Lack of fit test								
Sum of square	10.51	79.76	10.51	79.76	1.54	58.72	58.37	1.69
Df	3	3	3	3	3	6	6	3
Mean squares	3.50	26.59	3.50	26.59	0.51	9.79	9.73	0.56
F value	73.00	25.25	73.00	25.25	256.1	2.93	24.56	3.29
CV %	0.62	0.55	0.62	0.55	3.21	3.63	9.75	0.74
*R*^2^	0.919	0.944	–	–	0.96	0.849	0.84	0.99
Adequate precision value	10.99	5.50	–	–	21.92	11.12	10.539	42.84

### The optimum value for extraction parameters

The optimum values of extraction parameters for maximum predicted yield and antioxidant activity are shown in Table 4. Using these extraction parameters, we have validated the prediction model. The experimental value (Table 5) indicated 95–115% validity of the predicted model.

**Table 5.** Optimized parameters and predicted dependent variables for extraction of selected plant materials.

	*B. diffusa*	*R. emodi*	*N. lucifera*	*C. nurula*
Extraction parameters				
Extraction time (h)	5.05	7.00	6.43	5.35
Drug: Solvent (*v/v*)	11.99	6.32	10.68	11.79
Alcohol: Water (*v/v*)	58.24	67.97	68.99	68.99
Predicted value (Average ± SE mean)				
% Yield	19.26 ± 1.79	30.73 ± 1.86	15.24 ± 0.75	16.37 ± 0.35
Antioxidant values	86.25 ± 1.22	81.59 ± 0.47	74.16 ± 2.84	83.46 ± 2.00
Experimental values at optimized parameters (Average ± SE mean)				
% Yield	22.34 ± 2.84	29.78 ± 2.11	18.25 ± 1.45	17.25 ± 0.89
Antioxidant values	85.41 ± 4.56	81.65 ± 1.47	75.65 ± 1.35	81.56 ± 1.65

### Development of polyherbal combinations and their *in vitro* evaluation

**Figure 4.**
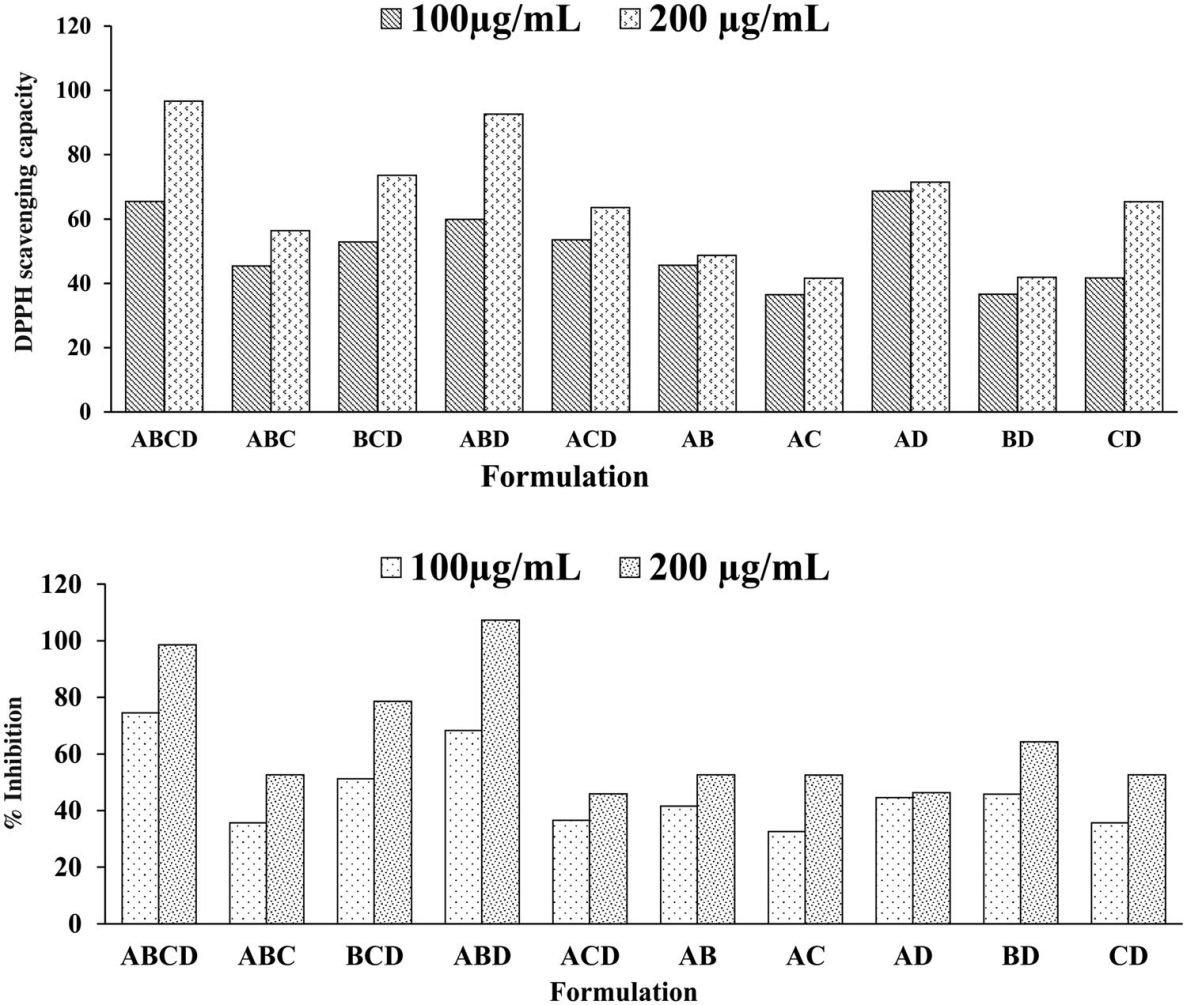
Screening of different combinations of extracts for (a) antioxidant activity and (b) XO inhibition potential.

Randomization has been widely accepted to reduce the risk of error considerably. Blocking reduces known but irrelevant sources of variation between conditions, thus allow a more precise estimation of the real causes of the variation. A total of 10 combinations were formulated by combining four optimized extracts and were screened *in vitro* for their antioxidant activity and xanthine oxidase inhibition potential. The results of antioxidant activity and XO inhibition potential of these combinations have been shown in Figure 4. Combination ABCD showed better XO inhibition potential followed by ABD as compared to other combinations. Thus, these two combinations were selected for further *in vivo* evaluation.

### Effect of polyherbal combination on biochemical parameters

The impact of selected combinations at two different doses on serum kidney function markers was assessed and shown in Table 6. Methotrexate treatment caused elevation of kidney function markers, and these were significantly reversed by treatment with combinations. Among two different doses administered to nephrotoxic rats, the level of reversal was more in higher doses as compared to a lower dose. But the efficacy of combination with combining four plant extracts (ABCD) was better as compared to a combination with three extracts (ABD). Kidney function markers, especially urea, uric acid, creatinine and albumin, were significantly altered in combination-treated rats as compared to methotrexate-treated rats. The effect of polyherbal combinations on kidney function markers of normal rats (sham control group) was also assessed. But no significant changes were observed as compared to normal control, which indicates that extract can improve the biochemical parameters only in nephrotoxin challenged rats and not the normal rats.

**Table 6.** Effect of polyherbal combinations on kidney function parameters.^a^

KFR parameters	Normal control	ABCD Sham	ABD Sham	Toxic control	ABCD 1	ABCD 2	ABD 1	ABD 2	Positive control
Urea (mg/dL)	35.6 ± 0.21	41.5 ± 1.21	42.5 ± 1.21	84.1 ± 1.21^###^	52.2 ± 0.94**	41.5 ± 0.74***	59.5 ± 1.21*	54.3 ± 0.74**	69.1 ± 0.86*
Creatinine (mg/dL)	0.68 ± 0.11	0.75 ± 0.07	0.77 ± 0.05	1.59 ± 0.08^#^	1.31 ± 0.08*	0.89 ± 0.11*	1.44 ± 0.08^ns^	1.11 ± 0.01*	1.41 ± 0.02^ns^
Uric acid (mg/dL)	2.67 ± 0.09	2.87 ± 0.08	2.68 ± 0.54	4.24 ± 0.05^##^	3.26 ± 0.12*	2.24 ± 0.08*	3.19 ± 0.08^ns^	2.89 ± 0.04*	2.98 ± 0.21*
Total protein (g/dL)	5.54 ± 0.07	5.61 ± 0.14	5.58 ± 0.65	7.59 ± 0.45^#^	5.42 ± 0.11^ns^	5.11 ± 0.05^ns^	6.73 ± 0.45^ns^	6.12 ± 0.05^ns^	6.11 ± 0.31^ns^
Albumin (g/dL)	2.89 ± 0.04	2.88 ± 0.11	2.79 ± 0.07	3.69 ± 0.04^#^	3.24 ± 0.08^ns^	2.75 ± 0.04*	3.64 ± 0.06^ns^	2.89 ± 0.11*	2.88 ± 0.08*
Globulin (g/dL)	1.89 ± 0.06	1.94 ± 0.08	1.95 ± 0.04	3.72 ± 0.03^#^	3.35 ± 0.06^ns^	2.58 ± 0.06*	3.09 ± 0.05^ns^	2.89 ± 0.05*	2.11 ± 0.09*
Total bilirubin (mg/dL)	0.28 ± 0.04	0.31 ± 0.05	0.32 ± 0.05	0.34 ± 0.02^ns^	0.31 ± 0.04^ns^	0.29 ± 0.04^ns^	0.29 ± 0.02^ns^	0.21 ± 0.03*	0.29 ± 0.05^ns^
Direct Bilirubin (mg/dL)	0.17 ± 0.04	0.12 ± 0.02	0.15 ± 0.05	0.13 ± 0.02^ns^	0.15 ± 0.05^ns^	0.16 ± 0.07^ns^	0.12 ± 0.01^ns^	0.13 ± 0.04^ns^	0.08 ± 0.07*
Indirect bilirubin (mg/dL)	0.11 ± 0.03	0.19 ± 0.02	0.17 ± 0.04	0.21 ± 0.03^ns^	0.16 ± 0.04*	0.13 ± 0.05*	0.17 ± 0.06*	0.08 ± 0.04*	0.11 ± 0.04*

^a^Data were expressed as mean ± SEM. ^#^Indicates data were compared with normal control. *Data were compared with toxic control. ***p < 0.001; **p < 0.01; *p < 0.1; ^ns^*p* > 0.5; ^###^*p* < 0.001; ^##^*p* < 0.01; ^#^*p* < 0.1.

Oxidative stress was assessed by measuring aspartate transaminase, alanine transaminase, catalase, malonaldehyde and alkaline phosphatase in serum as well as in kidney homogenates. The level of oxidative markers in serum and kidney homogenates of normal rats, methotrexate-treated rats and methotrexate-induced nephrotoxic rats treated with combinations are shown in Table 7. Serum malondialdehyde was evaluated as an indicator of renal lipid peroxidation and catalase as a ROS indicator. Combinations significantly suppressed both lipid peroxidation and ROS production. Oxidative markers in both serum and kidney homogenates were increased significantly in methotrexate-treated rats as compared to normal rats. The treatment with both the combinations significantly reversed the elevated oxidative markers. However, no significant changes in oxidative markers of normal rats treated with combination were observed.

**Table 7.** Effect of polyherbal combinations on oxidative markers in serum and in kidney homogenate.^a^

Oxidative markers	Normal Control	ABCD Sham	ABD Sham	Toxic control	ABCD 1	ABCD 2	ABD 1	ABD 2	Positive control
Serum									
SGOT (U/L)	35.4 ± 1.21	36.7 ± 1.31	34.6 ± 0.08	76.9 ± 0.08***	48.2 ± 0.08^##^	39.4 ± 0.08^###^	54.6 ± 0.08^#^	42.5 ± 0.08^###^	41.5 ± 0.08^###^
SGPT (U/L)	21.2 ± 0.89	20.4 ± 0.78	21.9 ± 0.65	82.6 ± 1.24***	32.6 ± 0.12^###^	28.6 ± 0.74^###^	41.2 ± 0.15^###^	35.4 ± 0.36^###^	31.5 ± 0.61^###^
Alkaline Phosphatase (U/L)	78.5 ± 0.45	79.2 ± 1.35	77.4 ± 1.25	212.5 ± 2.15***	125.6 ± 1.2^###^	98.6 ± 0.74^###^	135.4 ± 1.3^###^	111.4 ± 1.1^###^	97.6 ± 0.74^###^
Catalase (µmol H_2_O_2_/L/min)	179.4 ± 1.5	176.5 ± 1.25	177.4 ± 1.5	255.6 ± 1.89***	198.6 ± 1.4^#^	184.6 ± 1.4^###^	201.4 ± 1.35^ns^	194.5 ± 1.4^#^	184.5 ± 1.2^###^
Malonaldehyde (µmol/mL)	1.8 ± 0.045	1.8 ± 0.08	1.7 ± 0.04	4.11 ± 0.04***	2.4 ± 0.08^#^	1.9 ± 0.05^##^	2.7 ± 0.06^ns^	2.1 ± 0.08^#^	1.9 ± 0.08^##^
Kidney homogenate									
SGOT (U/L)	35.4 ± 0.78	36.7 ± 1.26	34.6 ± 0.15	76.9 ± 0.87***	48.2 ± 1.25^##^	39.4 ± 0.46^###^	54.6 ± 0.11^#^	42.5 ± 0.16^##^	41.5 ± 0.65^##^
SGPT (U/L)	21.2 ± 0.44	20.4 ± 0.54	21.9 ± 0.48	82.6 ± 0.69***	32.6 ± 0.78^###^	28.6 ± 0.25^###^	41.2 ± 0.21^###^	35.4 ± 0.65^###^	31.5 ± 0.54^###^
Alkaline Phosphatase (U/L)	78.5 ± 1.12	79.2 ± 1.12	77.4 ± 1.56	212.5 ± 1.45***	125.6 ± 0.9^##^	98.6 ± 1.24^###^	135.4 ± 1.23^##^	111.4 ± 1.2^###^	97.6 ± 1.36^###^
Catalase (µmol H_2_O_2_/g tissue/min)	79.4 ± 1.45	78.5 ± 1.56	79.4 ± 0.89	135.6 ± 1.48**	94.5 ± 0.78^#^	95.6 ± 0.98^#^	105.6 ± 1.24^##^	97.5 ± 0.68^#^	88.6 ± 1.47^##^
MDA (µmol/g wet tissue)	0.67 ± 0.03	0.68 ± 0.02	0.71 ± 0.03	2.8 ± 0.02*	1.4 ± 0.06^#^	0.91 ± 0.04^#^	1.78 ± 0.04^ns^	1.1 ± 0.03	0.96 ± 0.03^#^

^a^Data were expressed as mean ± SEM. *Data were compared with normal control. ^#^Data were compared with toxic control. ***p < 0.001; **p < 0.01; *p < 0.1, ^ns^*p* > 0.5; ^###^*p* < 0.001; ^##^*p* < 0.01; ^#^*p* < 0.1.

We have tested the ROS level in kidney homogenate. The level of ROS was remarkably increased (300%) in methotrexate-induced nephrotoxic rats as compared to normal rats. Upon treatment with polyherbal combinations and Neeri KFT, the ROS level was significantly lowered. In the case of rats treated with ABCD and ABD, the ROS level was 57.14 and 36.21%, respectively. Thus, the combinations could significantly (*p* < 0.01) ameliorate the ROS level, which supports the previous claims about the plant extract that can improve oxidative stress and prevent oxidative damage (Banerjee et al. 2018).

**Figure 5.**
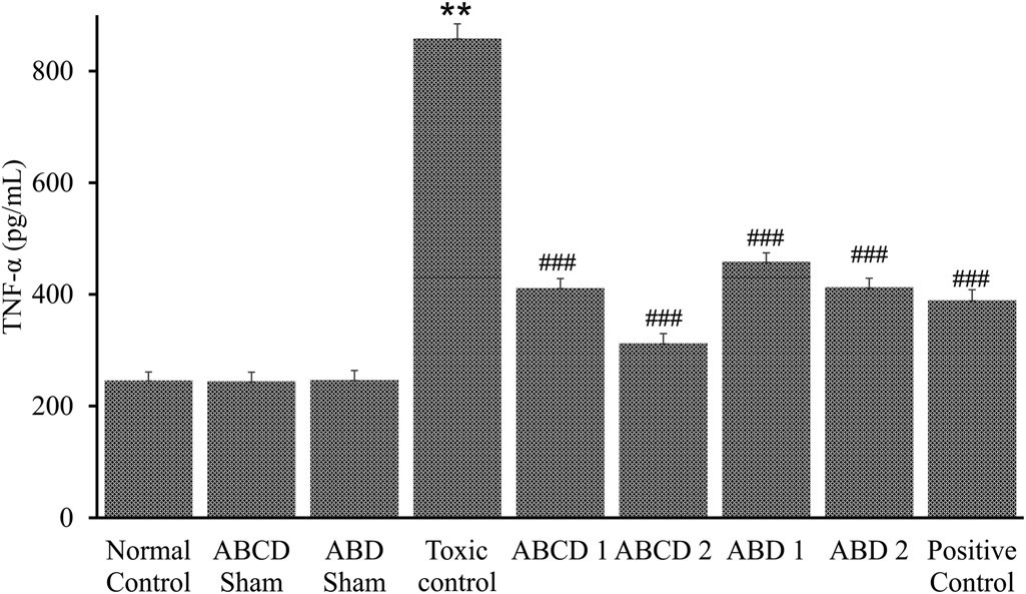
Effect of combination on TNF-α of methotrexate-induced nephrotoxicity in rats. Data were expressed as mean ± SEM. *Data were compared with normal control. ^#^Data were compared with toxic control. ***p < 0.001.

The inflammatory mediator, TNF-α, was also assessed in different groups. It is a crucial player in the inflammatory response during methotrexate-induced nephrotoxicity. Polyherbal combination treatment significantly decreased the elevation of TNF-α levels in comparison to the methotrexate intoxicated group (Figure 5). This study emphasizes a critical role for TNF-α in mounting the inflammatory response during methotrexate nephrotoxicity and the ensuing kidney tissue damage and acute renal failure.

### Effect of polyherbal combination on histology of kidney

**Figure 6.**
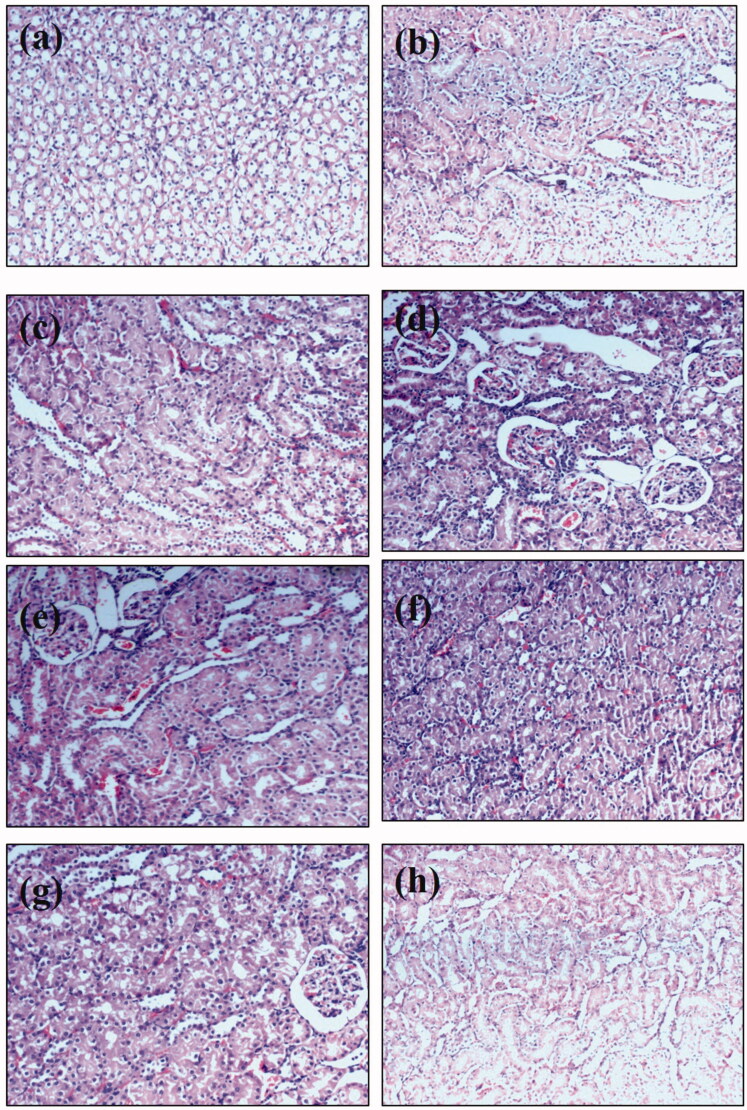
Microscopic images of kidney sections under light microscope (40×) after haematoxylin and eosin staining from animals of (a) normal control, (b) Sham control of combination ABCD, (c) Sham control of combination ABD, (d) methotrexate treated animals, (e) nephrotoxic rats treated with ABCD at a dose of 200 mg/kg, (f) nephrotoxic rats treated with ABCD rats at a dose of 300 mg/kg, (g) nephrotoxic rat treated with ABD combination at a dose of 250 mg/kg and (h) nephrotoxic rats treated with Neeri KFT tablet at a dose of 100 mg/kg.

To support the outcomes of the renal function markers, histopathological modifications were examined. Both control and combination-treated groups showed a typical histological pattern. Histopathological examination revealed that injection of methotrexate produced degeneration of renal tubules that showed cystic luminal dilatation as compared to the control group. Administration of both combinations at different doses was able to reverse methotrexate-induced histopathological damage (Table 8; Figure 6). The level of peritubular desquamation reversal in nephrotoxic rats treated with combinations was lower as compared to other histological features.

**Table 8.** Effect of formulation histological parameters of methotrexate induced nephrotoxic rats.

Histological features	Normal control	ABCD Sham	ABD Sham	Toxic control	ABCD 1	ABCD 2	ABD 1	ABD 2	Positive control
Glomerular congestion	−	−	−	+++	+	−	−	−	−
Tubular casts	−	−	−	+++	+	−	+	+	−
Peritubular desquamation	−	−	−	+++	++	+	+	−	+
Epithelial desquamation	−	−	−	+++	+	−	++	+	+
Blood vessel congestion	−	−	−	+++	−	−	−	−	−
Interstitial oedema	−	−	−	+++	+	−	−	−	−
Inflammatory cells	−	−	−	+++	+	−	+	−	−

### *In silico* screening

**Figure 7.**
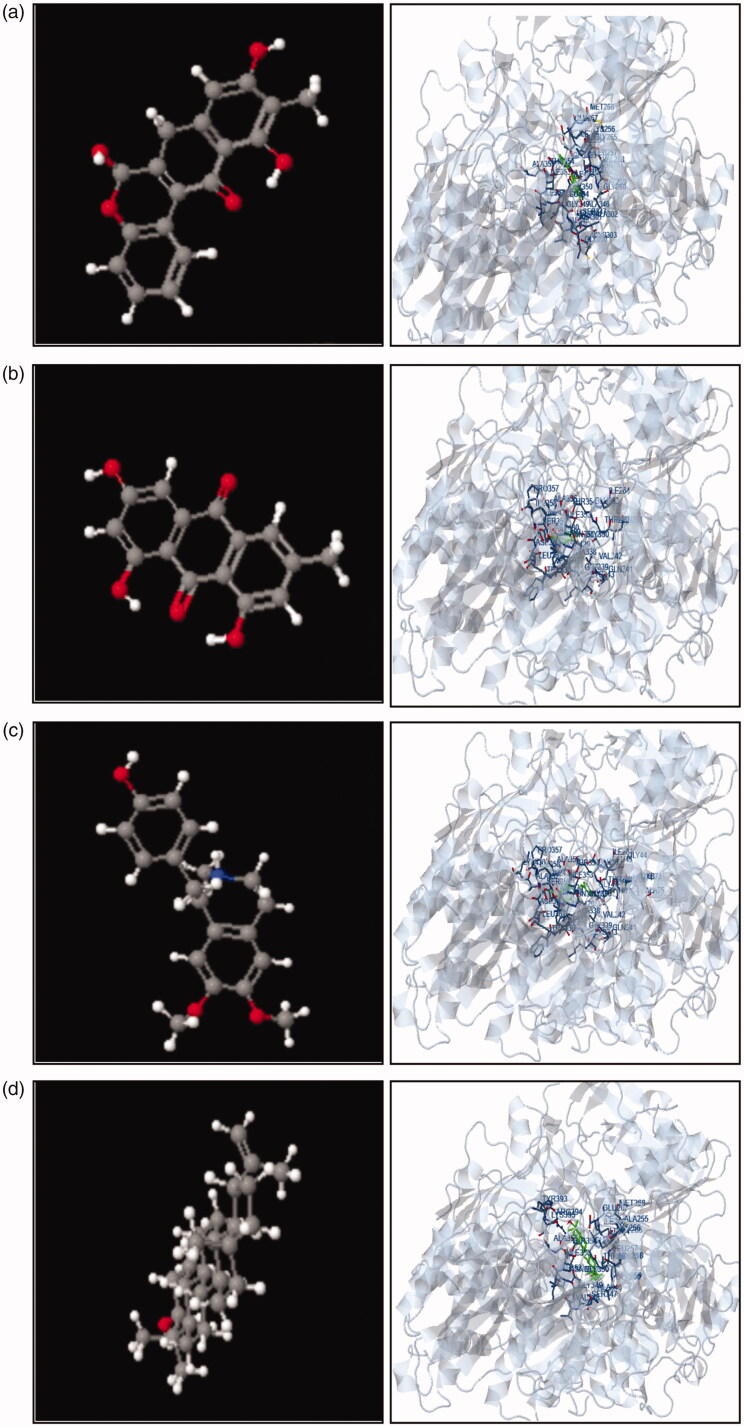
Three-dimensional interaction of XO proteins with (a) Boeravinone b, (b) emodin, (c) armepavine and (b) lupeol.

*In silico* screening, studies help to predict the extent of ligand-receptor interaction based on the binding energies or fitness score. In the present study, the docking of tested metabolites with xanthine oxidase was performed and the corresponding fitness score was < −5 kcal/mol. The highest fitness scored metabolites were further subjected to compare their interaction surface and total intermolecular energy with targeted molecules and proteins separately. We have tried four ligands (Boeravinone b, emodin, lupeol and armepavine) for protein-ligand interaction in docking studies. All these metabolites revealed a high fitness score (< −5 kcal/mol). Interaction of protein and ligand are shown in Figure 7. The docking studies revealed a good agreement with previous studies (Prathapan et al. 2014; Bhattacharjee et al. 2015; Ding et al. 2015). These studies presented the probable mechanism of action of nephroprotective activity by the polyherbal combination. Further, molecular-based research is required for confirmation of this above-proposed mechanism.

## Discussion

Nephrotoxicity signifies a condition of renal dysfunction that arises due to exposure to environmental chemicals or drugs. Oxidative stress and inflammation are the main pathological pathways involved in its aetiology, among others. A multifactorial condition like nephrotoxicity demands for a multipronged treatment modality for its mitigations. In this context, our current study aimed to develop a polyherbal combination for the amelioration of drug-induced nephrotoxicity. Four traditionally used Indian Medicinal plants were selected for this study. The extracts were characterized through their total phenolic content, flavonoid content and HPLC analysis. The parameters to maximize extraction efficiency were optimized using RSM. The different combinations were developed by randomized block design and screened for their XO inhibition potential and antioxidant activity. Out of these, the two best combinations were selected and tested against methotrexate-induced nephrotoxicity in rats to generate scientific data in support of *in vitro* results and their traditional claims.

The antioxidant potential of plant extracts was examined using DPPH free radical scavenging assay as this method is the most widely used, acceptable and simple method. The antioxidant molecule present in plant extract transforms DPPH radical into a stable molecule by transforming hydrogen atom and or electron from its atom. This steady conversion resulted in a decrease in absorbance, and it indicates the scavenging potential of analytes (Kedare and Singh 2011). Extracts of *B. diffusa*, *R. emodi*, *N. nucifera* and *C. nurvala* showed potent DPPH radical scavenging activity with half maximal effective concentration (EC_50_) close to that of reference ascorbic acid. The lower EC_50_ value of each plant extract indicates their higher antioxidant potential. Moreover, the reducing power of these plant extracts was increased when we increased the concentration of extract.

Phenols are essential plant constituents. A direct connection exists between complete phenolic content and plant species' antioxidant activity. It is mainly attributed to the scavenging ability of their phenolic hydroxyl groups that are reported to be effective hydrogen donors, making them excellent antioxidants (Vinson et al. 2001). Thus, it was rational and valuable to determine the total phenolic content of the selected plant extracts. These selected plant extracts showed good antioxidant activity and proved to also have higher phenol content. The results indicated that there was a positive and parallel relationship between phenol content and antioxidant activity. Several studies have shown that phenolic compounds in herbs, vegetables and fruits are a significant antioxidant component, and their antioxidant activity and complete phenolic content are directly related. Cai et al. (2004) also found a linear correlation between the content of total phenolic compounds and antioxidant capacity of several plant species. Phenolic compounds, including phenolic acid and flavonoids, can be enriched in aqueous extract (Dudonne et al. 2009). The extracts and their combinations showed potent DPPH scavanging potential. However, the extract may contain other metabolites also, and these can also contribute to antioxidant activity (Belyagoubi-Benhammou et al. 2019). The antioxidant activity of herbal extract could also be associated to its flavonoid content.

Flavonoids act as scavengers of various oxidizing species, i.e., hydroxyl radicals, superoxide anion and peroxy radicals. However, flavonoid quenches of singlet oxygen also (Ratty and Das 1988). Among the studied plant extracts, there was variation in total flavonoid content. It was highest in *B. diffusa* [(168.48 ± 24.75) mg rutin/g dry extract wt.)] and lowest in *R. emodi* [(132.48 ± 14.40) mg rutin/g dry extract wt.]. Flavonoids are polyphenolic compounds that are abundant and commonly found in nature. Flavonoids are categorized according to their chemical structure as flavones, isoflavones, flavonols, flavanones, chalcones, catechins and anthocyanidins. The pharmacological activities of flavonoids are closely related to their functional groups present in their structures.

Extraction time, drug-solvent ratio (% *w/v*) and alcohol-water ratio (% *v/v*) are essential factors in getting maximum yield with optimum therapeutic potential. The extraction parameters have greatly influenced the yield value of the extract as well as the biological activities. The results indicated that the maximum yield with the optimum antioxidant activity of *B. diffusa* was obtained in 5 h, the solvent was 12 times higher solvent ratio and 60% alcohol. Similarly, for *R. emodi,* the optimized parameters was 7 h, six times higher solvent ratio and 70% alcohol. Parameters of extraction were ranging from 5–7 h as extraction time, 6–12 times solvent, and 58–70% alcohol were the best for maximum yield with optimum antioxidant activities for these plant materials.

The 3 D response surface and the 2 D contour plots described by the regression model are drawn to illustrate the effect of the independent variables and interactive effects of each independent variable on the response variables. The shape of the respective contour plots shows whether or not there are essential mutual interactions between the independent variables. The elliptical character of the contour plots shows that interactions were important between the independent variables. The ideal values and the corresponding response variables could be predicted by the 3 D response surface plots and the corresponding contour plots and each variable pair could interact. The maximum predicted value is designated by the surface-confined in the smallest ellipse in the contour diagram. The disturbance plots show how the response changes as each factor move with all other factors at the reference value from the chosen reference point.

Polyherbal combination provides treatment of diseases in a holistic approach. Drug combination in Ayurveda is based on two principles, i.e., use as a single drug and use of more than one medicine, the latter known as a mixture of polyherbal formula. This important traditional therapeutic herbal approach deploys the use of a combination of several medicinal herbs to accomplish desired therapeutic efficacy and it is usually known as polyherbalism or polypharmacy (Parasuraman et al. 2014). The Ayurvedic literature Sarangdhar Samhita highlighted the concept of polyherbalism to achieve greater therapeutic efficacy (Srivastava et al. 2012). To obtain the desired therapeutic impact, the active phytochemical constituents of individual plants are inadequate. Although the active phytochemical components of individual plants have been well identified, they generally occur in a lower amount and are always insufficient to attain the desirable therapeutic impact. Scientific studies have disclosed that when mixed, these drugs of variable potency can theoretically generate a higher outcome compared to the plant’s actual use as well as the sum of its impact. In our study, a combination with the combination of four extracts (Combination: ABCD) showed better nephroprotective activity as compared to a combination containing three plant extracts (Combination: ABD). For both the combinations, a higher dose showed better activity as compared to a lower dose. The reversal of kidney function markers, oxidative stress marker and histological changes produced upon methotrexate treatment was also higher in combination ABCD as compared to combination ABD. The pharmacological activity of a combination of four plants was significantly higher as compared to the combination of these plant extracts. This beneficial herbal interaction phenomenon is known as synergism of pharmacodynamics effect (Spinella 2002). Some pharmacological activities of bioactive components of medicinal plants are only valuable when potentiated by those of other plants, but not apparent when used alone.

Scientific progress has led to the improvements of Ayurvedic polyherbal formulaions (PHFs) by studying multiple phytoconstituents and discovering beneficial combinations of herbs that work synergistically to generate a desirable result. Today, because of comparable efficacy, fewer side effects and better acceptability than allopathic drugs, the ‘renaissance’ of Ayurvedic PHFs is witnessed worldwide. They generate adequate impact and safety most of the moment, making them one of the drugs of choice that are extremely chosen. However, the insufficient understanding and misconception of the public about the safety of polyherbal formulation can lead to adverse effects, toxicity and unwanted interaction. The irresponsibility of manufacturers and inadequate regulatory control around the globe has also impacted the quality of produced herbal formulations, which can be hazardous to the health of customers. In this context, to decrease the hazardous risks, implement a good practice of strict regulatory control and public education on the proper use of PHFs, preventive and corrective action is much more essential. In this context, quality control analysis of any extract or herbal combination is necessary. In our study, we performed a chromatographic analysis of all extract to authenticate and to maintain its quality. HPLC fingerprinting analysis of combination was carried out for quality control analysis for its safety and efficacy. Similar chromatographic profiles ensure similar pharmacological activities. Boeravionone b (R_t_ 11.44), emodin (R_t_ 6.415), quercetin (R_t_ 8.407) and lupeol (R_t_ 16.181) are some common and specific markers of selected plant material were identified in the developed combination. The identification of these markers can be used for the pharmacokinetic profiling of combination. We have performed *in silico* screening of some specific markers with XO. These markers showed a higher affinity towards the selected protein, and this study supports its nephroprotective activity. Further, molecular research is needed to confirm its mechanism.

Oxidative stress is the primary parameter that plays a crucial role in the development of methotrexate nephrotoxicity and thus several parameters associated with oxidative stress parameters were assessed in our study. Our developed polyherbal combination does ameliorate oxidative markers. Previous reports of reduction in the oxidative markers with *B. diffusa* (Pareta et al. 2011b), *R. emodi* (Chang and Kim 2018), *N. nucifera* (Krishnamoorthy et al. 2009) and *C. nurvala* (Shirwaikar et al. 2004) are in line with the current findings. The antioxidant effect of the constituent ingredients seems to contribute to the overall nephroprotective action of the polyherbal combination.

The levels of TNF-α in the serum were increased after methotrexate injection. An increase in serum TNF-α was reported earlier in a model of nephrotoxicity (Morsy et al. 2013). The elevated renal expression of TNF-α appears to play a pathogenic role in methotrexate nephrotoxicity. Taken together, these results provide strong support for the view that TNF-α is an essential component in the mechanism of nephrotoxicity. Recent reports suggest that TNF-α may play a role in acute renal ischaemia (Harken et al. 2017) and endotoxemic injury (Knotek et al. 2001). Inhibition of TNF-α action, either with a TNF-binding protein or neutralizing antibodies reduced ischaemic injury in rats (Harken et al. 2017). We note that the TNF-α inhibitors reduced but did not wholly prevent nephrotoxicity. Further studies will be necessary to conclude if the measurement of serum or urine TNF-α levels may have value in identifying individuals with early methotrexate nephrotoxicity. Inhibition of TNF-α release or TNF-α activity afforded protection from methotrexate nephrotoxicity.

## Conclusions

A chromatographically characterized polyherbal combination from *B. diffusa*, *R. emodi*, *N. nucifera* and *C. nurvala* was developed. The developed polyherbal combination showed significant antioxidant and XO inhibition potential in *in-vitro* assays and strong nephroprotective activity in methotrexate intoxicated rats. The developed polyherbal combination can be explored further for the management of drug-induced nephrotoxicity and other chronic kidney diseases.
